# *In Silico* Genome-Wide Analysis of the ATP-Binding Cassette Transporter Gene Family in Soybean (*Glycine max* L.) and Their Expression Profiling

**DOI:** 10.1155/2019/8150523

**Published:** 2019-01-10

**Authors:** Awdhesh Kumar Mishra, Jinhee Choi, Muhammad Fazle Rabbee, Kwang-Hyun Baek

**Affiliations:** Department of Biotechnology, Yeungnam University, Gyeongsan, Gyeongbuk 38541, Republic of Korea

## Abstract

ATP-binding cassette (ABC) transporters constitute one of the largest gene families in all living organisms, most of which mediate transport across biological membranes by hydrolyzing ATP. However, detailed studies of ABC transporter genes in the important oil crop, soybean, are still lacking. In the present study, we carried out genome-wide identification and phylogenetic and transcriptional analyses of the ABC gene family in* G*.* max*. A total of 261* G*.* max* ABC (GmABCs) genes were identified and unevenly localized onto 20 chromosomes. Referring to protein-domain orientation and phylogeny, the GmABC family could be classified into eight (ABCA-ABCG and ABCI) subfamilies and ABCG were the most abundantly present. Further, investigation of whole genome duplication (WGD) signifies the role of segmental duplication in the expansion of the ABC transporter gene family in soybean. The Ka/Ks ratio indicates that several duplicated genes are governed by intense purifying selection during evolution. In addition,* in silico* expression analysis based on RNA-sequence using publicly available database revealed that ABC transporters are differentially expressed in tissues and developmental stages and in dehydration. Overall, we provide an extensive overview of the GmABC transporter gene family and it promises the primary basis for the study in development and response to dehydration tolerance.

## 1. Introduction

Active transport mechanisms require the intake of energy and can be operated by either of two mechanisms: (1) free energy change associated with ATP hydrolysis (primary transport), or (2) assisted by the potential energy of the chemical gradient of another molecule (secondary transport). Depending on structural homology and transport mechanism, primary transporters are subdivided into three classes — rotary motor ATPases (F-, V-, and A-ATPases), P-type ATPases, and a large family of integral membrane proteins named ATP-binding cassette (ABC) transporters [1]. The ABC transporters represent one of the largest families of membrane proteins, ubiquitously found within the kingdoms of eukarya, eubacteria, and archaea [1–3]. These proteins employ ATP hydrolysis to transport various substrates (e.g., heavy metals, endogenous metabolites, inorganic anions, drugs, lipids, sterols, hormones, amino acids, peptides, vitamins, and sugars) in and out of cells across biological membranes [2, 4]. Primarily, plant ABCs transporters are recognized only in detoxification processes [5]. Afterwards, an abundant number of plant ABCs have been characterized in physiological and developmental processes. So far, more than 100 ABC transporters encoding genes have been characterized across plant genome. The numbers of plant ABC transporters are more than those of most other organisms, suggestive of their involvement in a broad range of biological functions [6, 7]. The Arabidopsis genome contains 131 ABC genes, whereas 121 ABC genes were reported in the rice genome [8, 9]. For instance, white brown complex (WBC) homolog, pleiotropic drug resistance (PDR), multidrug resistance (MDR), and MDR-associated protein (MRP) are best-exemplified subfamilies among plant ABC transporters. However, genome-wide surveys and expression analysis of this gene family have not been performed in* G*.* max*.

ABC transporters are a family of membrane-bound proteins that mediate transport across biological membranes by hydrolyzing ATP. They contain conserved cytoplasmic domains termed as nucleotide-binding domains (NBD) (also referred to as ATP-binding cassettes). These NBD are ~200 amino acids coupled with ATPase and often serve as energy suppliers for substrate translocation as well as nontransport processes [10]. Apart from this domain, ABC proteins also consist of one or two hydrophobic transmembrane domains (TMDs). Based on the transporter class, each TMD comprises 6-10 transmembrane *α*-helices (frequently 6 helices present among most of the exporters). TMDs determine substrate specificity and are responsible for the translocation of a substrate across the lipid bilayer [8, 11]. The NBDs hydrolyze ATP and drive conformational changes in the attached TMDs, thus allowing substrates to cross the lipid bilayer of the membrane and be either imported into or exported out of the cytoplasm. Further, members of the ABC superfamily are classified into exporters, importers, and nontransporting proteins. Exporter and importer types of ABC proteins comprise at least two TMDs and two NBDs. Exporters are present in all kingdoms and importers solely in bacteria and plants [12]. Hence, coming to sequence comparison, there are 3 classes of ABC systems present among the last common ancestors (archaea, bacteria, and eukarya). Class 1 comprises ABC proteins in which TMDs and NBDs are fused to form a single polypeptide (exporters) (found in eukaryotes), class 2 includes nontransport ABCs lacking TMDs (nontransporters; found in both eukaryotes and prokaryotes), and class 3 includes mainly transporters with NBDs and TMDs formed by separate polypeptide chains (canonical importers; exclusively found in prokaryotes) and some bacterial exporters [13, 14].

According to sequence similarity, ABC transporter proteins are classified according to structure (full, half, or quarter molecule) and orientation (forward or reverse). Three structural types are defined as follows. A full transporter comprises two TMDs along with two NBDs and can be either in forward (TMD1-NBD1-TMD2-NBD2) or in reverse (NBD1-TMD1-NBD2-TMD2) orientation. Half transporters are composed of one TMD and one NBD and can be in forward (TMD1-NBD1) or reverse orientation (NBD1-TMD1). They usually constitute a feasible unit by forming a virtual full transporter as homo- or heterodimers [15, 16]. The third type of transporter has no TMDs, but two NBDs [17]. Owing to the lack of TMD, they are not usually involved in the transmembrane transport mechanism. Few transporters consist of single NBDs and are similar to prokaryotic ABC proteins, referred to as quarter transporters. The protein is either a half-size or full-size transporter, but the majority of subfamilies comprise both types of transporters [17, 18].

NBD is present among all three structural types and includes several highly conserved motifs within the domain: Walker A (also called a phosphate-binding loop), Walker B, Q-loop, D-loop, switch H-loop, and a signature motif or C motif (LSGGQ) [19]. The major function of D-loop is holding dimers together such as the switch H-loop interacting with the transmembrane domain. Both Walker motifs A and B form the P-loop, which binds to ATP, and, at last, the Q-loop and H-loop include special residues crucial for interacting with the *γ*-phosphate of the ATP [13]. Furthermore, signature motifs exclusively found in ABC proteins make them discernible from another type of ATPase [20, 21].

Human Genome Organization (HUGO) scheme has been presently accepted and well documented for categorization of human as well as plant ABC proteins [22]. In accordance with eukaryotes, eight basic subfamilies (A–H) identified based on the domain organization and homologous relationship. However, ABCH subfamily has been reported in arthropod genomes and absent in fungi, mammals, and plants [23, 24], apart from another prokaryotic-like ABC protein with a single NBD domain called subfamily I. They are highly soluble and found only in plants but are absent in most animal genomes [17]. Hence, in total, nine subfamilies (ABCA-ABCI) have been identified, and eight of them (ABCA-ABCG and ABCI) are present in plant genomes [6]. Furthermore, proteins which belong to ABCA-ABCD subfamilies have a forward direction for domain organization (TMD-NBD) whereas ABCG and ABCH subfamilies have reverse domain organization (NBD-TMD). Proteins of ABCE and ABCF include only two NBDs and are designated as soluble proteins. They do not participate in any transport-related processes (but their NBDs can cluster with those of other ABC proteins). ABCI proteins possess only one single domain, predominantly NBD, rendering them difficult to identify and categorize. This genetic fact points out that some of ABCI proteins can bind together into systemic multisubunit ABC transporters and this subfamily is also designated as soluble proteins. Considering similar domain organization, ABCI subfamily transporters are evolved due to the movement of genes from mitochondria and plastids to the nucleus [7, 17, 18].

Proteins of the ABCA subfamily possess several half-size (ABC2 homolog, named ATH) variants and, so far, one full size (ABC1 homolog, named AOH) has been reported. Full-size variants are likely associated with the transportation of sterols, whereas the function of half-size ABC2 homologs remains unclear. ABCB subfamily possesses both full size (MDR; also known as p-glycoprotein) and half size such as transporter associated with antigen processing (TAP) and ABC transporter of the mitochondria (ATM). Full-size variants of ABCB proteins are known for detoxification and transport of auxins. Half-size ABCB is involved in heavy metal tolerance. Typically ABCC subfamily proteins are full-size ABC transporters and called MDR-associated protein. They are thought to be associated with the transportation of organic anions and xenobiotic anions into the vacuole. Few half-size ABCC are also reported in Arabidopsis and rice. ABCD subfamily proteins are generally half-size (peroxisomal membrane protein) transporters. Some exceptionally full-size ABCD variants are found in Arabidopsis and* Vitis vinifera*. They are implicated in the transport of fatty acids into the peroxisome. ABCG subfamily has both full- and half-size variants. Full-length members, also referred to as PDR transporters, have been implicated in heavy metal resistance and auxin transport [8, 25, 26]. WBC transporters are half-length members of the ABCG subfamily and requirements for the export of cuticular lipids and alkanes [27, 28]. Both ABCE and ABCF proteins are extremely conserved among all living organisms during evolution. ABCE are also referred to as RNase L inhibitors (RLI), associated with ribosome biogenesis. In most eukaryotes including human, single ABCE have been identified [29]. ABCF subfamily is conventionally known as the general control nonrepressible (GCN) subfamily and is found to be involved in stress tolerance in plants [18]. ABCI subfamily transporters have single NBD domain. A few well-studied examples of ABCI subfamily are closely associated with iron-sulfur center biogenesis complex and cytochrome C maturation complex [7, 17, 18]. Moreover, previous studies revealed that the ABCB, ABCC, and ABCG subfamilies possess a relatively higher number of genes in the plant, in comparison to other eukaryotes such as human and yeast [17].

Soybean [*Glycine max* (L.) Merr.] is one of the most frequently cultivated crops in the world and belongs to the Leguminosae subfamily. Seeds are an important source of human food, cooking oil, and animal feed, because of their abundant protein and oil content. Soybean plants also play a significant role in soil fertility, as they fix atmospheric nitrogen during symbiosis. In addition, they are the predominant source of isoflavonoids and play a significant role in human diet and health [30]. The first draft genome of soybean (*Glycine*.* max* var. Williams 82) was reported in 2010 [31], and the latest version of the genome assembly, version 2.0 (*G. max *Wm82.a2.v1), contains 56,044 protein-coding genes; yet their functional contribution to crop traits remains mostly unidentified. A large number of soybean coding genes are presently annotated for Gene Ontology biological process (GOBP) terms with experimental evidence. In this study, we screened the new soybean genome database (*G*.* Max *Wm82.a2.v1; https://phytozome.jgi.doe.gov/pz/portal.html) for ABC transporter genes. We performed a detailed analysis of their classification, chromosome distribution, physicochemical properties, phylogenetic analysis, and duplication. Finally, we verified the differential expression profiles of all GmABC genes in nine different* G*.* max* tissues, seven developmental stages, and various stress conditions. This study provides important information about the origin and evolution of the GmABC family in* G*.* max* and lays the foundation for further studies of the functions of this gene family.

## 2. Materials and Methods

### 2.1. Identification of ABC Transporter Genes in Soybean

There are different types of putative ABC proteins such as ABC transporter domain (PF00005), the ABC-2 transporter domain (PF01061), the ABC transporter transmembrane region domain (PF00664), the CYT domain (PF01458), or the mce-related protein domain (PF02470). The Hidden Markov Model (HMM) profiles of all the abovementioned proteins were downloaded from the Pfam 27.0 database (http://pfam.xfam.org/; [32]). Proteomes of soybean were searched against all the abovementioned HMM profiles to detect all ABC transporter domains with an E-value cut-off < 1E^−4^ in the new soybean genome database (*Glycine max* Wm82.a2.v1) derived from phytozomev12 (https://phytozome.jgi.doe.gov/pz/portal.html; [33]), with default settings. In addition, corresponding genomic and coding sequences along with their chromosomal positions were also retrieved from Phytozome. Based on Verrier et al. [17], identification and classification of these ABC proteins were performed.

The protein sequences retrieved from the above approaches were collected together. Further redundancy of sequences was eliminated in order to generate a unique set of putative ABC transporter proteins. Additionally, the presence of ABC transporter domains in all these proteins was confirmed by domain search via SMART (http://smart.embl-heidelberg.de/; [34]) and PROSITE (https://prosite.expasy.org/scanprosite/; [35]). Physicochemical properties such as molecular weight (Mw) and isoelectric point (pI) for each GmABC protein were calculated using the ExPASy proteomics server (https://web.expasy.org/compute_pi/; [36].

### 2.2. Chromosomal Distribution, Gene Structure Prediction, and Gene Duplications in GmABCs

The chromosomal position of all GmABC genes was retrieved from* G*.* Max *Wm82.a2.v1 genome database in Phytozome using BLASTN searches. MapChart 2.3 (Wageningen UR, Wageningen, the Netherlands; [37]) was used to map the ABC transporter genes onto chromosomes from short-arm to long-arm telomeres, in ascending order of physical position (bps). Tandem duplications of ABC transporter genes in* G. max* were identified in Plant Tandem Duplicated Genes Database (PTGBase) (http://ocri-genomics.org/PTGBase/; [38]. Further, GmABCs in duplicated genomic regions were obtained for syntenic mapping from the batch download option of Plant Genome Duplication Database (PGDD; http://chibba.agtec.uga.edu/duplication/; [39]. Synonymous substitution (Ks) and nonsynonymous substitution (Ka) rates for each segmentally duplicated GmABCs were retrieved. Subsequently, the period of duplication incidents in soybean was predicted according to the equation T=Ks/2*λ*; here the mean synonymous substitution rate (*λ*) was taken as 6.1*∗*10^−9^ [40, 41]. The numbers and positions of exons and introns within GmABC gene were detected by comparing the full-length cDNA sequences with the corresponding genomic DNA sequences using an online tool: gene structure display server (http://gsds.cbi.pku.edu.cn/index.php; [42].

### 2.3. Phylogenetic Tree Analysis and Functional Classification of ABC Proteins

Multiple sequence alignments were executed with the deduced amino acid sequences of GmABCs by using ClustalW [43]. Afterwards, a phylogenetic tree was constructed using the Maximum-Likelihood (ML) [44] method provided in the Molecular Evolutionary Genetics Analysis (MEGA) 6.0 software tools [45] based on the Jones, Taylor, and Thornton (JTT) matrix-based model. The accuracy of an inferred tree was checked with bootstrap analysis (with 1,000 replications).

### 2.4. Gene Ontology Annotation and In Silico Expression Analysis through RNA-Sequencing Data

The Gene Ontology (GO) analysis of soybean ABC transporters was performed using the Blast2GO program (https://www.blast2go.com/; [46]. The full-length amino acid sequences of GmABC proteins were uploaded to the program for the blast, followed by mapping and annotation. The analysis was carried out in three categories: biological process, molecular function, and cellular component.

We downloaded the RNA-seq data of nine different soybean tissues and analyzed the expression levels of all 261 GmABC genes based on their fragments per kilobase of transcript per million mapped reads (FPKM) values. FPKM values for each GmABC were computed to assess the transcript level of GmABCs across nine tissue genes and the data were normalized across tissues. The heatmap for GmABC genes was generated using TIGR Multi-Experiment Viewer (MeV4) software package [47]. To obtain expression patterns of GmABCs in different development stages and perturbation, we have used the publicly available mRNA sequence data from Genevestigator V3 database (https://genevestigator.com/gv/doc/intro_plant.jsp; [48]. We downloaded the program from the Genevestigator homepage and used academic license for analysis in the local computer. Using plant biology tool, we first identified 238 probes for development and perturbation conditional search for all 261 GmABC genes. Seven different developmental stages of soybean are germination, main shoot growth, inflorescence formation, flowering, fruiting, bean development, and final ripening. In the case of perturbation, light intensity, germination, dehydration, and ozone irradiation are included for transcript data analysis. All data represent the log2 ratio of relative expression pattern.

### 2.5. Homology Modeling for Three-Dimensional Structure

All the GmABC proteins were investigated against the Protein Data Bank (PDB) [49] by BLASTP (with the default parameters) to recognize the best template having a similar sequence and known three-dimensional structure. Further, three-dimensional (3D) structures of GmABCs were generated by intensive protein modeling using Phyre2 server (Protein Homology/AnalogY Recognition Engine; http://www.sbg.bio.ic.ac.uk/phyre2; [50]). The intensive mode uses the multitemplate alignment of experimentally solved protein structures. In addition, it incorporates ab initio folding simulation termed as Poing [51] to model regions of proteins with no apparent homology to known structures.

### 2.6. Cis-Elements Analysis

The upstream sequences of the GmABCs, which were 2 kb upstream from the translation start site, were retrieved from Phytozome. These sequences were analyzed for the identification of regulatory* cis*-elements important for gene expression under abiotic stress, development, and hormone signaling using PlantPAN 2.0 (http://plantpan2.itps.ncku.edu.tw/; [52].

## 3. Results

### 3.1. Identification of GmABC Transporter Genes and Their Physicochemical Properties

We identified 261 proteins with ABC transporter domains in the* G*.* max* genome. Different transcripts of the same gene were not considered. Among 261 GmABCs, there were 114 full transporters, 116 half transporters, and 31 soluble putative ABC proteins. ABCA, ABCB, ABCC, ABCD, and ABCG subfamilies contain full transporters, as well as half transporters. As in most eukaryotes, soybean has only one ABCE gene [53]. The details of all 261 soybean ABC transporter proteins, including gene identifier, genomic location, domain organization, description, protein length, molecular weight, isoelectric point (pI), and their subfamily, are listed in Table S1. The lengths of ABC transporter proteins range from 67 (Glyma.16G125700) to 1826 (Glyma.03G136000) amino acids, specifying that there are huge variations within the soybean ABC transporter gene family. The predicted molecular weights of GmABC proteins ranged from 7.72 to 203.97 kDa and the pI values were between 4.41 and 10.5.

### 3.2. Chromosomal Distribution, Gene Structure, and Gene Duplication of GmABCs

**Figure 1:**
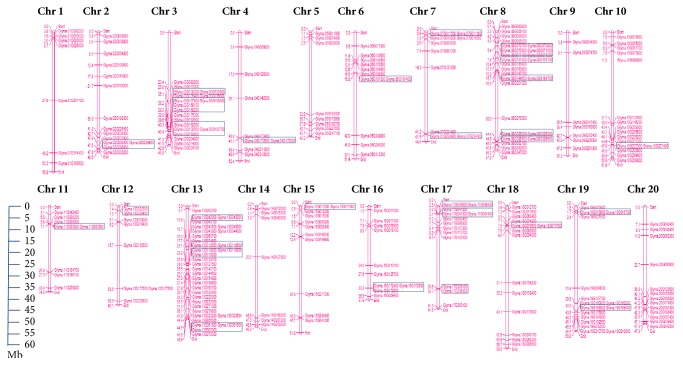
**Graphical representations of locations for putative ABC transporter genes on each soybean chromosome.** Genes within the rectangle box are putatively tandem duplicated. The chromosome size is indicated by its relative length using the information from Phytozome. The scale (in megabase) on the left depicts the relative lengths of the different chromosome in* G. max*. The gene names are depicted on the right side of each chromosome corresponding to the position of each gene. The figure was produced using the MapChart program. One GmABC could not be anchored onto any specific chromosome.

As was previously mentioned, there are more than double ABC transporters present in soybean as compared to Arabidopsis or rice and that was likely due to recent WGD in soybean. Concerning gene duplication, it includes segmental/WGD and tandem duplication (TD) events [54]. To evaluate the expansion of the GmABC transporter gene family, a chromosome map was constructed based on the locations provided by Phytozome 12 (*G*.* max* Wm82.a2.v1). The 261 GmABC transporter genes are unevenly distributed throughout all 20 soybean chromosomes (2n=40, Figure 1). The largest number of GmABCs was found on chromosome 13 (34 genes), followed by chromosome 08 (26 genes) and chromosome 03 (19 genes). Both chromosomes 10 and 19 comprise 16 GmABCs each, whereas one gene could not be anchored onto any specific chromosome. It signifies that each subfamily of GmABCs was also unevenly distributed. Gene structures of the 261 soybean ABC transporters reveal high complexity with intron number ranging from 1 to 38 (Figure S1). The detailed chromosomal locations of GmABC genes and the number of introns in the gene are shown in Table S1.

Generally, tandem duplication is defined as two paralogs separated by less than five genes in the same chromosome. For detection of tandem duplication, we investigated tandem arrays of 261 GmABCs using PTGBase. Tandem arrays are described as multiple members of genes appearing inside the same or neighboring intergenic regions. We detected 28 GmABC clusters containing 69 tandemly duplicated genes (genes are depicted in a rectangle box in Figure 1 and arrays are shown in Table S2), which were identified on 15 chromosomes. Gene duplications are considered a prime factor in the evolution of genomes and expansion of gene families [55, 56]. Recent studies indicated that soybean has undergone two WGD events (approximately 59 and 13 million years ago), which resulted in ~75 % paralogous gene pairs [31, 57, 58]. Polyploidy introduces immense duplications, ultimately leading to the vital source of genetic innovation [59, 60]. Here, we identified a total of 190 duplicated gene pairs within 261 GmABC genes by using the Plant Genome Duplication Database (Table S3). The nonsynonymous/synonymous substitution ratio (Ka/Ks) explains the selective evolutionary pressure acting on a gene. The Ka/Ks ratio for majority (94.74%) of the gene pairs was found < 1, recommending that most of GmABCs are evolved under purifying selection with limited functional divergence after duplication (as shown in Table S3, the Ka/Ks value of most of the duplicated gene pairs ranged from 0.021 to 0.682). The Ka/Ks value of ten pairs of duplicated genes was more than 1, which signifies positive selection pressure. Thus, it concluded that segmental along with tandem duplication was involved in the expansion of the ABC transporter gene family in the soybean genome [61–63].

### 3.3. Phylogenetic Analysis of Soybean ABC Transporter Genes

**Figure 2:**
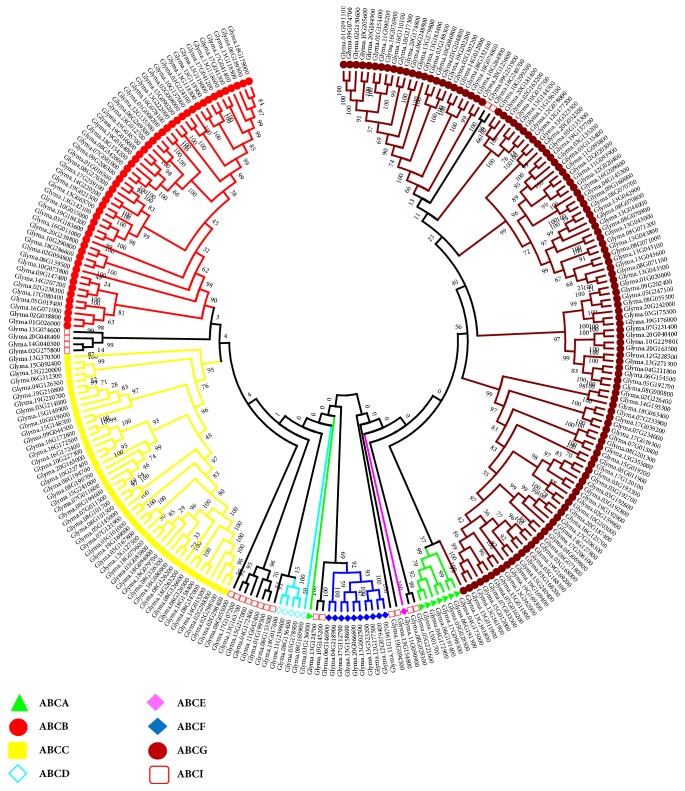
**Phylogenetic relationship of ABC transporter gene family proteins in soybean.** The phylogenetic tree was built using the ML method implemented in MEGA6.0 based on full-length amino acid sequences. The evolutionary history was inferred based on the JTT matrix-based model. The numbers at the nodes represent bootstrap percentage values based on 1000 replications. Genes from each subfamily are indicated with different bullet point colors.

To elucidate the evolutionary relationships of soybean ABC transporter genes, an unrooted phylogenetic analysis was constructed from all 261 GmABC proteins using ML methods with 1,000 bootstraps. Genes from each subfamily (ABCA-ABCG and ABCI) are depicted with different bullet point colors (Figure 2). Referring to the ML phylogenetic tree analysis, the ABC family is grouped into eight subfamilies. Among ABCG, ABCB, and ABCC were the largest subfamilies containing 117, 52, and 48 members, respectively, accounting for a total of 83% of all GmABC members. ABCI, ABCF, ABCA, and ABCD included 20, 10, 8, and 5 members, respectively. There is only one member in ABCE subfamily identified (Table S1). All subfamilies were clustered together in the phylogenetic tree, except ABCI. In fact, ABCI genes were scattered throughout the tree. Our analysis corroborates previous studies, which indicated that the ABCG subfamily has the highest number of members and is accountable for the expansion of this gene family in comparison to other eukaryotes [7, 64].

### 3.4. In Silico Expression Analysis of GmABCs

#### 3.4.1. Expression Analysis of GmABC Genes Exhibits a Differential Pattern in Tissues

**Figure 3:**
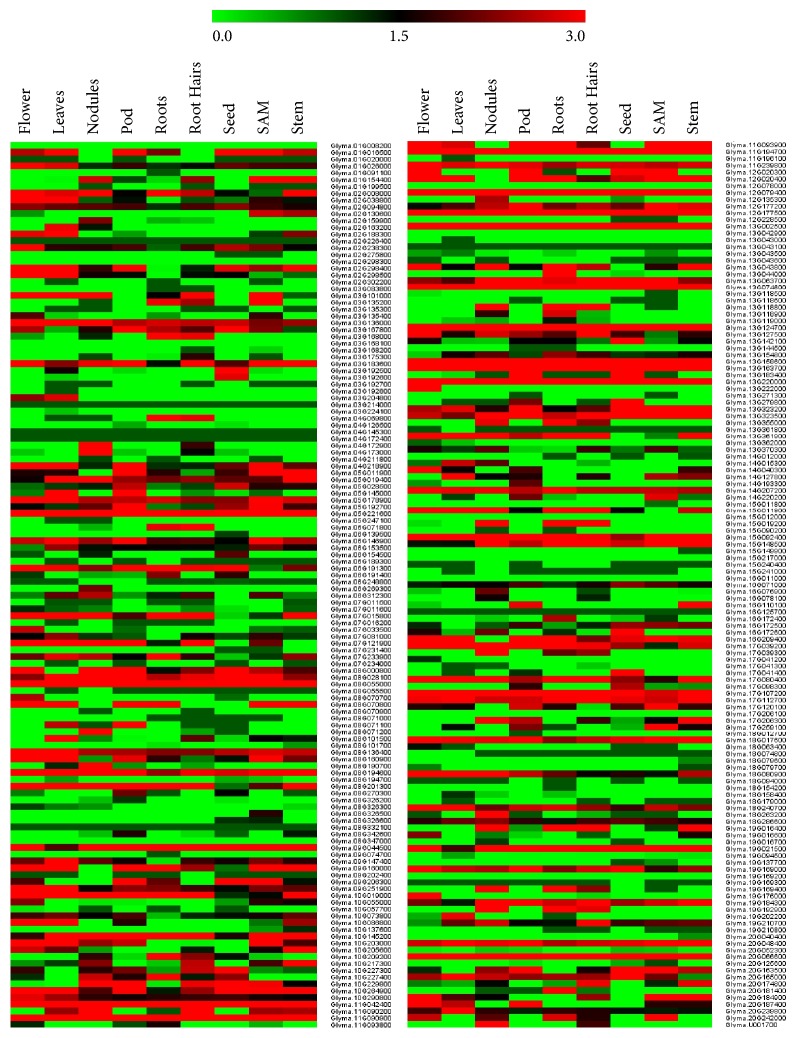
**Expression profiles of 261 soybean ABC transporter genes in nine different tissues derived from RNA-seq data of Phytozome database.** FPKM values of GmABC transporters genes were transformed by log2, and the heatmap was constructed with MeV 4. Expression levels are illustrated by graded color scale, representing relative expression pattern ranging from 0 (downregulated) to 3 (upregulated). Red, green, and black represent positive, negative, and zero, respectively. Gene IDs are given on the top. Genes with similar profiles across arrays are grouped on top by hierarchical clustering method.

RNA-sequencing is a robust tool for exploring the transcription patterns of certain genes using high-throughput sequencing approach. Expression levels of all 261 GmABC genes based on their FPKM values analyzed in nine different tissues and heatmap of all genes displayed differential tissue-specific expression patterns (Figure 3). Noticeably, thirty-one genes showed higher-level of transcript accumulation in all the tissues under this study suggesting that these have a unique role. They belong to all class of ABC subfamily, but, among these genes, most belong to soluble type ABC transporters (7 ABCF, 7 ABCI, and 1 ABCE) followed by 4 ABCD. Above all, 46 GmABC genes in pods, 40 genes in flower, 37 genes in leaf, 23 genes in the stem, 27 genes in shoot apical meristem, 41 genes in the root, and 17 in nodule accumulated the highest level of transcripts. Expressions of 20 genes were found to be lowest in all tissues.

#### 3.4.2. GmABC Genes Showed Diverse Levels of Expression at Various Developmental Stages

Plant development is a very complex phenomenon that altered the expression of a number of genes to meet physiological and metabolic demand. To investigate the developmental regulation of GmABC transcripts, expression of genes was checked at seven different developmental stages, namely, germination, main shoot growth, inflorescence formation, flowering, fruit formation, bean development, and final ripening. Expression of all these GmABC genes was investigated using Genevestigator based mRNA-seq data. Among 261 GmABC genes, 43 GmABC showed the highest level of expression at all the developmental stages of soybean (Figure S2A). About 37 genes were not detected in any of the seven development stages.

#### 3.4.3. Expression Patterns of the GmABC Genes in Response to Abiotic Stress and Light and during Germination

The Genevestigator analysis showed expression of all 261 GmABC genes based on mRNA-sequence data. Based on the analysis, it was observed that twenty genes were highly expressed in all three dehydration study conditions (6 hr., 12 hr., and 24 hr.) in two soybean cultivar Benning and PI-416937. Thus, they might have a role in dehydration tolerance in plants. More than 40 genes were dominantly expressed during germination in soybean cultivar Williams (Figure S2B). Remarkably, almost all genes were found to be unexpressed in both light intensity condition (mature and immature leaf) as well as in ozone study. Overall, the analyses revealed that GmABCs highest level of expression in dehydration suggests dehydration regulatory function in soybean.

### 3.5. Gene Ontology Annotations

**Figure 4:**
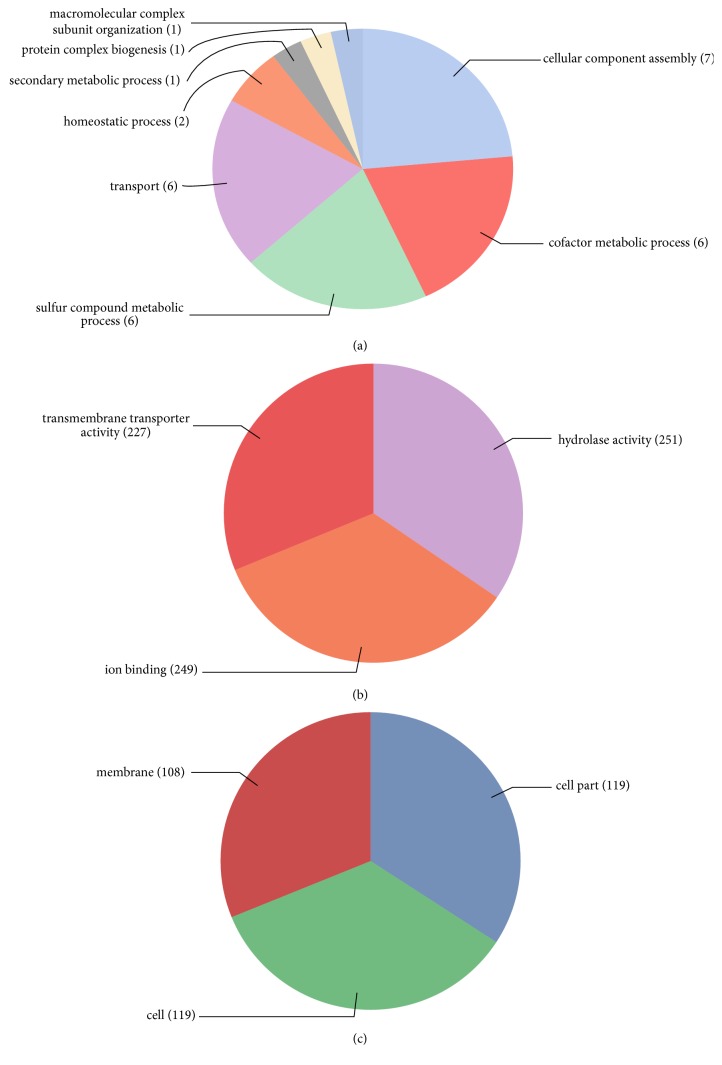
**GO annotation of GmABC transporter proteins.** The annotation was performed on three categories: (a) biological process, (b) molecular function, and (c) cellular component.

The GO slim analysis conducted using Blast2GO and result showed the putative participation of GmABCs proteins in diverse biological processes (Figure 4). The majority of proteins were involved in transport, cellular component assembly, and metabolic process. Cellular localization prediction showed that GmABC proteins are equally localized in the cell membrane and in the nucleus.

### 3.6. Homology Modeling

**Figure 5:**
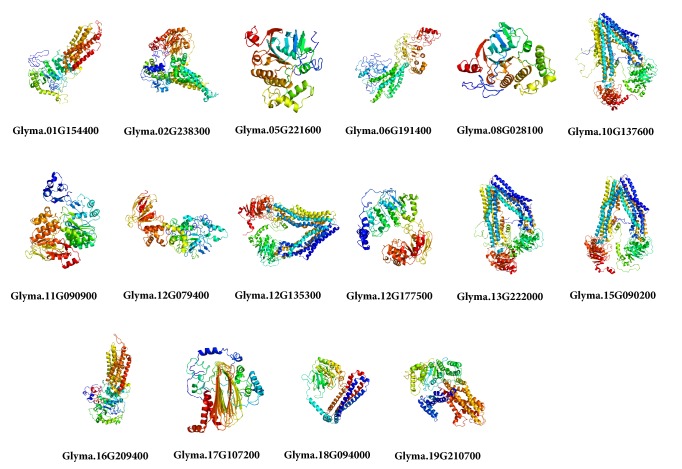
Predicted structures of 16 GmABC proteins.

Sixteen ABCs proteins having higher homology were selected and, consequently, Phyre2 was used to predict the homology modeling (Figure 5). Remarkably, these 16 proteins belong to diverse subfamily. The protein structure of all the GmABCs is modelled at >90% confidence (% residue varied from 71 to 99; Table S4). All the predicted protein structures are considered highly reliable and offer a preliminary base towards understanding the molecular function of GmABC proteins.

### 3.7. Cis-Regulatory Elements in the Promoters of Soybean ABC Genes

Expression of the gene can be regulated at various levels, such as transcription and posttranslational modification level.* Cis*-regulatory elements are present in the upstream region and specifically control the gene expression in higher plants [65]. Two kb promoter regions for each of the 261 GmABCs were retrieved. Then, we applied the PlantPan2.0 website to analyze the* cis*-elements and total of 298 different* cis*-elements identified in all GmABCs. The analysis identified the presence of several stress-related, development and hormone inducible motifs, such as Homeo Box/ leucine Zipper, MBS (MYB Binding Site), WRKY-domain, NAC-domain, GT1consensus, dehydration responsive element binding factors (DREB), ABRE (Abscisic Acid-Responsive Element), GATA-box, AT-hook containing transcription factors, DNA binding with one finger (DOF), GCN-4, TC-rich, and Skn-1. Identified* cis*-regulatory elements are known to regulate various stress responses, developmental processes, and hormonal signaling. Further, they are found to be distributed randomly in both positive and negative strands of the promoters of these genes. A total of 13 common* cis*-regulatory elements were observed in all GmABCs and highly conserved among all genes (Table S5).

## 4. Discussion

In the present study, 261 ABC transporter genes were identified in the genome of soybean; among them, 31 members are recognized as soluble proteins without TMDs. Immense variation in size (from 298 to 49491 bps) and gene structure (variable introns number from 1 to 38) indicates divergence within the soybean ABC transporters gene family. The intron/exon organization also signifies their evolutionary history across gene families. The ABC transporter family has been extensively characterized in other plants, with 131 ABCs genes in Arabidopsis [17], 121 in rice [21, 66], 130 in* Zea mays *[67], 179* Brassica rapa *[68], 135 members in* Vitis vinifera *[69], and 154 members in tomato [70]. As compared with these plants,* G*.* max* contains more ABC transporter genes, which is not surprising since* G*.* max* is a paleopolyploid and has undergone two WGD events, as there is a significant number of segmental and tandem duplicated genes and they also contributed to the expansion of the gene family. Further, phylogenetic analysis revealed that 8 subfamilies, among all subfamilies, were clustered together, except ABCI. ABCI were clustered into different clades, suggesting more diverged gene members. Among 261 GmABC proteins, members of ABCG were most abundant followed by ABCB and ABCC. Our analysis corroborates with previous studies, which indicated that ABCG subfamily is the largest and is accountable for the expansion of this gene family in comparison to other eukaryotes [7, 64]. It has been reported that ABCG transporters are also concerned with the adaptation to the land environment [71].

To elucidate the significance of GmABCs gene, we have performed the* in silico *expression analysis in three aspects based on their transcript data: (1) tissue-specific, (2) development stage, (3) germination and stress condition. Tissue-specific expression patterns have been determined via FKPM data, while development and stress conditions are represented by m-RNA sequence data. There are 31 host genes (Table 1 and Figure 3), which were highly expressed in all nine tissues; among them, 15 belong to soluble ABC proteins.

These aforementioned genes may play a key role in tissue development. Furthermore, seven genes are exclusively expressed in nodules. Similarly, three genes are exclusively detected in flowers and have some important role in flower development. Correspondingly, Glyma.02G163200 and Glyma.19G202200 are only expressed in leaves and Glyma.13G044000 and Glyma.16G172400 are only expressed in roots. Glyma.06G154500, Glyma.13G142100, and Glyma.17G041400 are exclusively expressed in seeds, suggesting that they may play some roles in seed development. In addition, 20 genes were found to have no detectable level of expression values possible due to the very low abundance of the RNAs of these host genes.

Approximately 43 show expressions in all seven development stages and few have a high level of expression (shown in Table 2 and Figure S2A). Overall, the analysis revealed that the highest number of genes (~40 genes) was expressed during inflorescence formation stage, suggesting their role in inflorescence development. It is noteworthy that the expression of 37 host genes was not observed in any developmental stage; this may be due to inducible features of these host genes.

Some genes are expressed in the dehydration stage in cultivar Benning and PI-416937 (shown in Table 2 and Figure S2B). Hence, these ABC genes can play a potential role in dehydration tolerance mechanism. Most of these genes also possess stress-responsive* cis*-acting element in their promoter region. Analogously, at germination stage of some genes in cultivar Williams, few host genes showed higher level of expression (shown in Table 2 and Figure S2B). Still, more experimental evidence is required to find out the specific functions of the ABC transporter gene.

## 5. Conclusions

The ABC transporter gene family is one of the largest known proteins, ubiquitously present in all living organisms and playing a diverse function such as regulation of growth and resistance processes across the plant kingdom. In the present study, we conducted* in silico* genome-wide annotation of soybean ABC transporter gene family. In total, 261 putative ABC transporter genes were identified and these genes were located unevenly in 20 chromosomes. The number of genes was approximately double that in Arabidopsis and rice. Since segmental duplication, tandem duplication along with WGD might have contributed to the expansion of this gene family. Further, these proteins were classified into 8 key subfamilies and results of our phylogenetic analysis corroborated those of previous studies in another plant. Data files of the physicochemical properties of ABC transporter gene display great variation in gene structure, their length, and domain orientation. The GO analysis revealed multiple functions for the GmABC transporter proteins, including their role in transport and metabolic process. The RNA-seq and Genevestigator data illustrated that GmABC genes were expressed in various tissues, development stages, and the germination stage, as well as in response to dehydration stresses on the transcriptional level, signifying their diverse function. Besides,* cis-*regulatory element analysis of GmABCs also suggests their role in the development and various abiotic stresses. The results provide comprehensive information for the further functional study of the GmABC gene family.

## Figures and Tables

**Table 1:** List of highly expressed ABC transporter genes in different tissues.

**Expressed tissue**	**Genes**
All nine tissues	Glyma.03G136000, Glyma.05G178900, Glyma.05G221600, Glyma.08G028100,
	Glyma.08G055000, Glyma.08G136400, Glyma.08G194600, Glyma.09G044500,
	Glyma.11G042400, Glyma.11G090900, Glyma.11G194700, Glyma.11G239800,
	Glyma.12G079400, Glyma.13G002500, Glyma.13G063700, Glyma.13G074600,
	Glyma.13G124700, Glyma.13G158600, Glyma.13G163700, Glyma.13G220000,
	Glyma.13G323500, Glyma.14G207200, Glyma.15G092400, Glyma.17G080400,
	Glyma.17G107200, Glyma.17G112700, Glyma.18G017600, Glyma.19G021500,
	Glyma.19G169000, Glyma.20G048400, Glyma.20G066600

Only in Nodules	Glyma.02G159900, Glyma.04G172900, Glyma.04G173000, Glyma.06G269300, Glyma.08G071200, Glyma.12G135300, Glyma.U001700

Only in flowers	Glyma.07G033500, Glyma.08G070700, Glyma.13G222000

Only in leaves	Glyma.02G163200 Glyma.19G202200

Only in roots	Glyma.13G044000, Glyma.16G172400

Only in seeds	Glyma.06G154500, Glyma.13G142100, Glyma.17G041400

**Table 2:** List of GmABC transporter genes expressed during development stage, dehydration, and germination.

**Expression condition**	**Genes**
**Development stage**	Glyma.01G016500, Glyma.03G136000, Glyma.03G167800,
	Glyma.03G183600, Glyma.04G218900, Glyma.05G178900,
	Glyma.06G146900, Glyma.07G121900, Glyma.07G233900,
	Glyma.08G136400, Glyma.08G550000, Glyma.08G194600,
	Glyma.09G044500, Glyma.09G160000, Glyma.10G019000,
	Glyma.10G145200, Glyma.11G093900, Glyma.11G239800,
	Glyma.12G020400,Glyma.12G079400, Glyma.13G163700,
	Glyma.13G323200, Glyma.15G092400,Glyma.16G209400,
	Glyma.18G017600, Glyma.18G080900, Glyma.19G021500, Glyma.20G066600

**Dehydration**	Glyma.02G008000, Glyma.3G101000, Glyma.07G033500,
	Glyma.07G233900, Glyma.08G136400, Glyma.10G019000,
	Glyma.10G217300, Glyma.11G194700, Glyma.12G020400,
	Glyma.12G079400, Glyma.12G177500, Glyma.13G127500,
	Glyma.15G012000, Glyma.12G0192000,Glyma.16G076900,
	Glyma.16G110100, Glyma.17G039200, Glyma.17G041200,
	Glyma.17G112700, Glyma.20G174800

**Germination stage**	Glyma.01G016500, Glyma.01G026000, Glyma.02G038800,
	Glyma.02G0298400, Glyma.03G183600, Glyma.04G128900,
	Glyma.05G011900, Glyma.05G019400, Glyma.05G221600,
	Glyma.08G000800, Glyma.08G028100, Glyma.08G070700,
	Glyma.08G160900, Glyma.08G194700, Glyma.08G201300,
	Glyma.09G206300, Glyma.10G086800, Glyma.10G145200,
	Glyma.10G203000, Glyma.12G177200, Glyma.13G063700,
	Glyma.13G142100, Glyma.13G323200, Glyma.17G080400,
	Glyma.17G120100, Glyma.18G240700, Glyma.19G021500,
	Glyma.19G184300, Glyma.20G048400, Glyma.20G125600

## Data Availability

The data used to support the findings of this study are available from the corresponding author upon request.

## Supplementary Materials

Supplementary 1 FIGURE S1: Gene structure of soybean ABC transporter genes. Gene structures were made using online tool GSDS (Hu et al., 2015; http://gsds.cbi.pku.edu.cn/). As shown in the legend, blue bars stand for untranslated regions (UTR). One yellow bar represents an exon and one black string represents an intron. The scale on the bottom is in kilobase (kb).

Supplementary 2 FIGURE S2A: Hierarchical cluster of expression profiles of 261 soybean ABC transporter genes in seven different development stages derived from Genevestigator software (https://www.genevestigator.com/). Expression levels are illustrated by graded color scale, representing the log2 ratio of relative expression pattern ranging from 0 to 100%. For the maroon/white scale, all gene-level profiles were normalized for coloring such that, for each gene, the highest signal intensity obtains the value 100% (maroon), and absence of signal obtains the value 0% (white).

Supplementary 3 FIGURE S2B: Heatmap representation of 261 differentially expressed ABC transporter genes during perturbation derived from Genevestigator V3 (https://www.genevestigator.com/). Expression levels are illustrated by graded color scale, representing the log2 ratio of relative expression pattern ranging from -2.5 (downregulated) to 2.5 (upregulated). Red, green, and black represent positive, negative, and zero, respectively. Gene IDs are given on the top.

Supplementary 4 TABLE S1: List of GmABC transporter genes identified and their basic physical properties.

Supplementary 5 TABLE S2: The ratio of Ka/Ks and distribution of duplicated soybean ABC transporter genes in respective blocks as obtained from Plant Genome Duplication Database (PGDD).

Supplementary 6 TABLE S3: Tandemly duplicated soybean ABC transporter genes obtained from PTGBase database.

Supplementary 7 TABLE S4: Characteristics of 16 GmABC proteins selected for homology modeling.

Supplementary 8 TABLE S5: Common putative* cis*-elements identified in the promoter sequences of GmABCs.
