# Corrigendum to “*In Silico* Genome-Wide Analysis of the ATP-Binding Cassette Transporter Gene Family in Soybean (*Glycine max* L.) and Their Expression Profiling”

**DOI:** 10.1155/2019/1289174

**Published:** 2019-03-21

**Authors:** Awdhesh Kumar Mishra, Jinhee Choi, Muhammad Fazle Rabbee, Kwang-Hyun Baek

**Affiliations:** Department of Biotechnology, Yeungnam University, Gyeongsan, Gyeongbuk 38541, Republic of Korea

In the article titled “*In Silico* Genome-Wide Analysis of the ATP-Binding Cassette Transporter Gene Family in Soybean (*Glycine max* L.) and Their Expression Profiling” [1], the Acknowledgments section was incorrect. It should be corrected as follows:

This work was financially supported by Korea Institute of Planning and Evaluation for Technology in Food, Agriculture, Forestry and Fisheries (IPET) through Agri-Bio Industry Technology Development Program and financially supported by Ministry of Agriculture, Food and Rural Affairs (MAFRA) (117044-3).

## References

[1] Mishra A. K., Choi J., Rabbee M. F., Baek K.-H. (2019). *In silico* genome-wide analysis of the ATP-binding cassette transporter gene family in soybean (*glycine max* L.) and their expression profiling. *BioMed Research International*.

