# Supplementary figures and images for: In Silico Genome-Wide Analysis of the ATP-Binding Cassette Transporter Gene Family in Soybean (Glycine max L.) and Their Expression Profiling
Source: Biomed Res Int. 2019 Jan 10;2019:8150523. doi: 10.1155/2019/8150523 (PMC6350567; doi:10.1155/2019/8150523)

## Slide 1
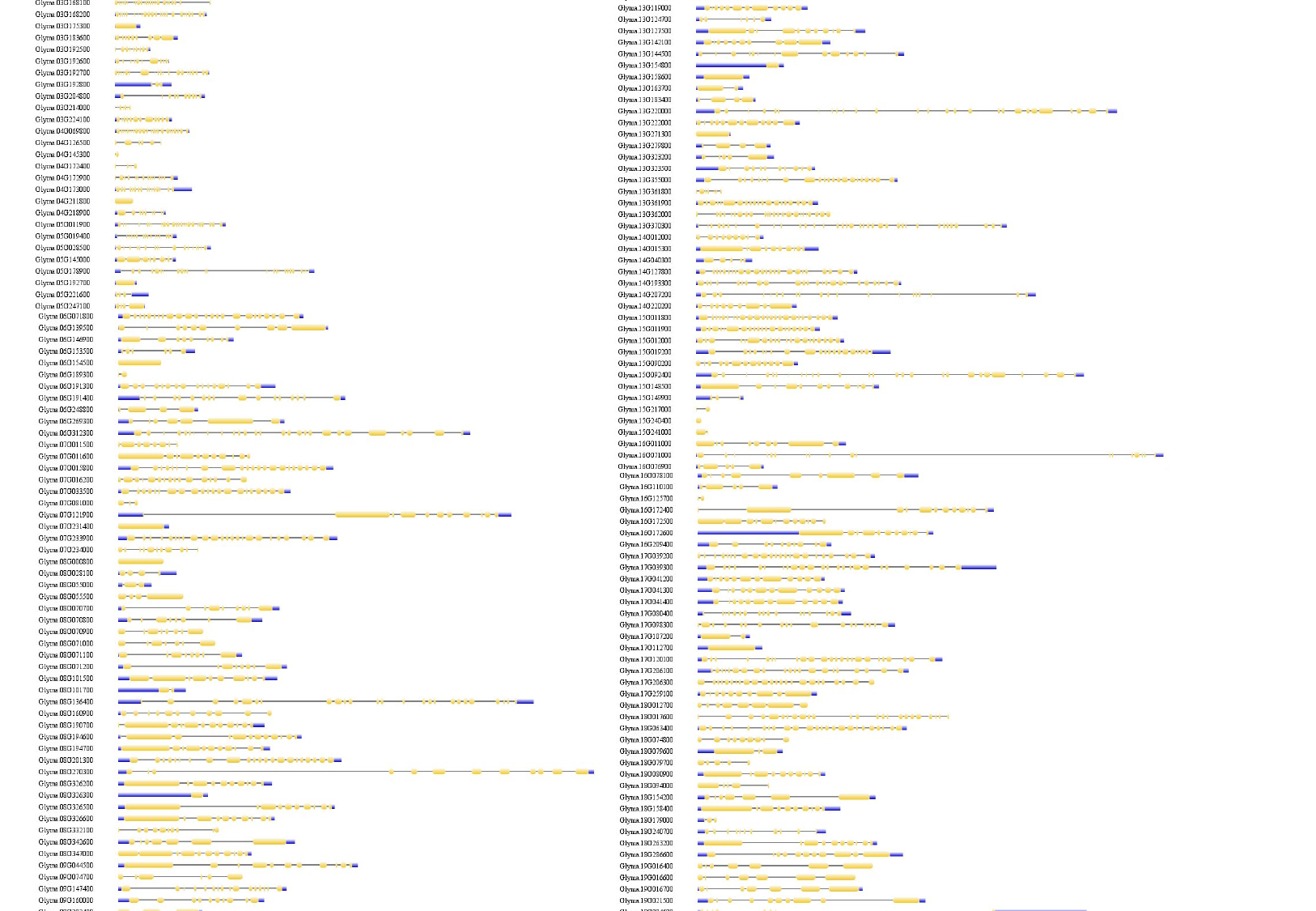

Exon
Upstream/ downstream
Intron

Supplement: Supplementary 1 — FIGURE S1: Gene structure of soybean ABC transporter genes. Gene structures were made using online tool GSDS (Hu et al., 2015; http://gsds.cbi.pku.edu.cn/). As shown in the legend, blue bars stand for untranslated regions (UTR). One yellow bar represents an exon and one black string represents an intron. The scale on the bottom is in kilobase (kb). [file 8150523.f1.pptx]

## Slide 1
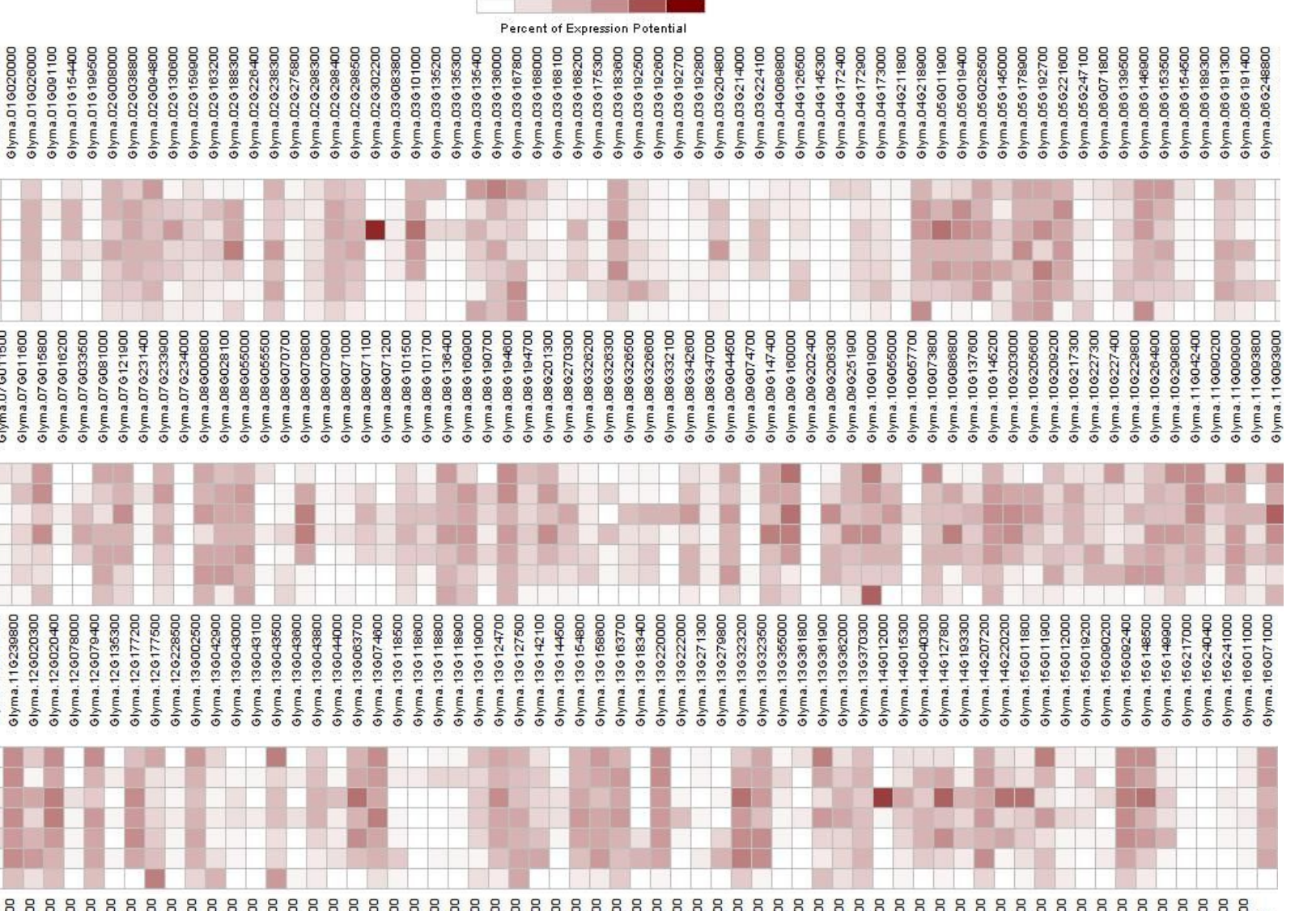

Supplement: Supplementary 2 — FIGURE S2A: Hierarchical cluster of expression profiles of 261 soybean ABC transporter genes in seven different development stages derived from Genevestigator software (https://www.genevestigator.com/). Expression levels are illustrated by graded color scale, representing the log2 ratio of relative expression pattern ranging from 0 to 100%. For the maroon/white scale, all gene-level profiles were normalized for coloring such that, for each gene, the highest signal intensity obtains the value 100% (maroon), and absence of signal obtains the value 0% (white). [file 8150523.f2.pptx]

## Slide 1
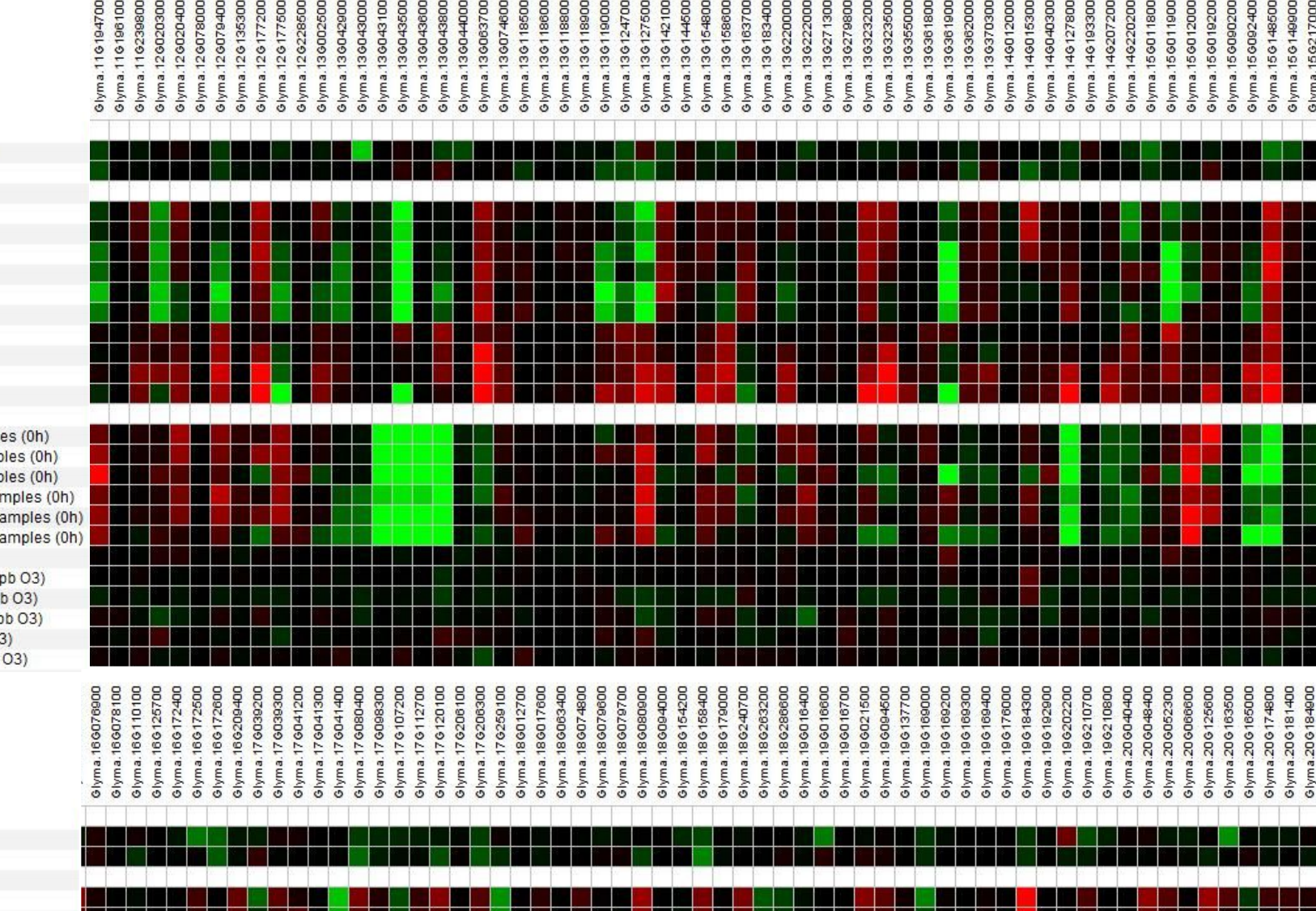

Supplement: Supplementary 3 — FIGURE S2B: Heatmap representation of 261 differentially expressed ABC transporter genes during perturbation derived from Genevestigator V3 (https://www.genevestigator.com/). Expression levels are illustrated by graded color scale, representing the log2 ratio of relative expression pattern ranging from -2.5 (downregulated) to 2.5 (upregulated). Red, green, and black represent positive, negative, and zero, respectively. Gene IDs are given on the top. [file 8150523.f3.pptx]
