# Supplementary material for: In Silico Genome-Wide Analysis of the ATP-Binding Cassette Transporter Gene Family in Soybean (Glycine max L.) and Their Expression Profiling
Source: Biomed Res Int. 2019 Jan 10;2019:8150523. doi: 10.1155/2019/8150523 (PMC6350567; doi:10.1155/2019/8150523)
Supplement: Supplementary 7 — TABLE S4: Characteristics of 16 GmABC proteins selected for homology modeling. [file 8150523.f7.doc]

TABLE S4. Characteristics of 16 candidate SiWD40 proteins chosen for homology modeling.

| **Protein** | **% of residues modelled** | **Confidence (%)** | **Subfamily** |
| --- | --- | --- | --- |
| Glyma.01G154400 | 97 | 90 | ABCG |
| Glyma.02G238300 | 96 | 90 | ABCB |
| Glyma.05G221600 | 93 | 90 | ABCI |
| Glyma.06G191400 | 71 | 90 | ABCA |
| Glyma.08G028100 | 85 | 90 | ABCI |
| Glyma.10G137600 | 95 | 90 | ABCB |
| Glyma.11G090900 | 99 | 90 | ABCE |
| Glyma.12G079400 | 92 | 90 | ABCF |
| Glyma.12G135300 | 96 | 90 | ABCB |
| Glyma.12G177500 | 95 | 90 | ABCF |
| Glyma.13G222000 | 96 | 90 | ABCB |
| Glyma.15G090200 | 97 | 90 | ABCB |
| Glyma.16G209400 | 92 | 90 | ABCG |
| Glyma.17G107200 | 73 | 90 | ABCI |
| Glyma.18G094000 | 98 | 90 | ABCC |
| Glyma.19G210700 | 86 | 100 | ABCC |
