# Supplementary material for: In Silico Genome-Wide Analysis of the ATP-Binding Cassette Transporter Gene Family in Soybean (Glycine max L.) and Their Expression Profiling
Source: Biomed Res Int. 2019 Jan 10;2019:8150523. doi: 10.1155/2019/8150523 (PMC6350567; doi:10.1155/2019/8150523)
Supplement: Supplementary 8 — TABLE S5: Common putative cis-elements identified in the promoter sequences of GmABCs. [file 8150523.f8.docx]

TABLE S5. Common putative *cis*-elements identified in the promoter sequences of GmABCs.

| ***Cis*-element** | **Signal sequence** | **Expression pattern** |
| --- | --- | --- |
| Homeodomain-ZIP | CAATNATTG | Developmental processes |
| WRKY | TGAC | GA, ABA, PR proteins, plant  defenses |
| DOFCOREZM | AAAG | Leaf, shoot, carbon metabolism |
| CAATBOX1 | CAAT | Seed |
| WBOXATNPR1 | TTGACY | Wound |
| NODCON2GM | CTCTT | Nodule |
| GATABOX | GATA | Leaf, shoot, light, molecular  light switches |
| GT1CONSENSUS | GRWAAW | Leaf, shoot, light, SA |
| GTGANTG10 | GTGA | Pollen |
| BOXCORE | GATAA | Leaf, shoot, light regulation |
| POLLEN1LELAT52 | AGAAA | Pollen |
| CACTFTPPCA1 | YACT | C4 plant, mesophyll |
| TAAAGSTKST1 | TAAAG | Guard cell, K+ influx channel |
